# 2374. Predicting the Public Health Impact of Bivalent Vaccines and Nirmatrelvir-Ritonavir against COVID-19

**DOI:** 10.1093/ofid/ofad500.1995

**Published:** 2023-11-27

**Authors:** Hailey J Park, Sophia T Tan, Tomás M León, Seema Jain, Robert Schechter, Nathan C Lo

**Affiliations:** University of California, San Francisco, San Francisco, California; University of California, San Francisco, San Francisco, California; California Department of Public Health, Richmond, California; California Department of Public Health, Richmond, California; California Department of Public Health, Richmond, California; University of California, San Francisco, San Francisco, California

## Abstract

**Background:**

Uptake of COVID-19 bivalent vaccines and nirmatrelvir-ritonavir (Paxlovid) remains low across the United States. The goal of this study is to predict the public health impact of increasing uptake of bivalent vaccines and nirmatrelvir-ritonavir in key risk groups on COVID-19 cases, hospitalizations, and deaths.

**Methods:**

We used a statistical model that was calibrated to person-level COVID-19 outcome data (cases, hospitalizations, deaths) from the California Department of Public Health, to predict the cumulative number of COVID-19 outcomes over the next six months. We modeled the impact of perfect uptake of these interventions in different risk groups defined by age (50+ years, 65+ years, 75+ years) and vaccination status (everyone, primary series only, previously vaccinated). We predicted the number of averted COVID-19 outcomes with each strategy by applying published estimates of vaccine and nirmatrelvir-ritonavir effectiveness; we computed number needed to treat (NNT).

**Results:**

The most efficient strategy (based on NNT) for averting severe COVID-19 was targeting interventions to the 75+ years age group. In the California population, perfect coverage of bivalent boosters in the 75+ years age group averted 3,920 hospitalizations (95% UI: 2,491-4,882; 7.8% total averted; NNT 387) and 1,074 deaths (95% UI: 774-1,355; 16.2% total averted; NNT 1,410). Perfect uptake of bivalent boosters in those 50+ years averted 7,778 hospitalizations (95% UI: 4,858-9,763; 15.4% total averted; NNT 1,144) and 1,630 deaths (95% UI: 1,134-2,122; 24.6% total averted; NNT 5,458). Perfect uptake of nirmatrelvir-ritonavir in the 75+ years age group averted 5,644 hospitalizations (95% UI: 3,947-6,826; 11.2% total averted; NNT 11) and 1,669 deaths (95% UI: 1,053-2,038; 25.2% total averted; NNT 35), while perfect uptake in the 50+ years age group averted 9,699 hospitalizations (95% UI: 6,882-11,611; 19.2% total averted; NNT 19) and 2,323 deaths (95% UI: 1,458-2,866; 35.0% total averted; NNT 79).

**Figure:**
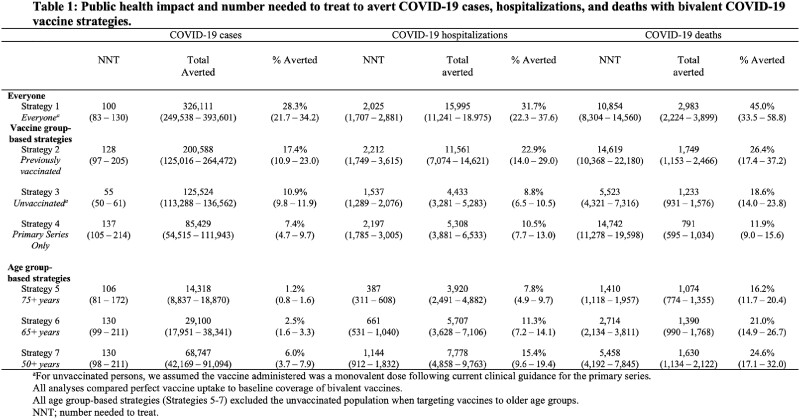

**Figure:**
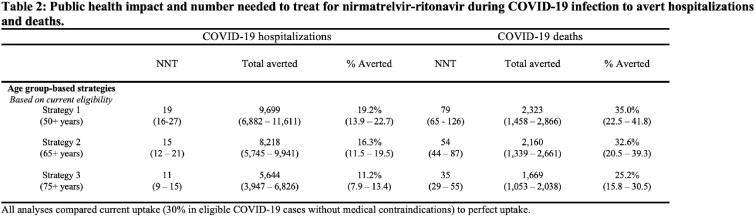

**Conclusion:**

Prioritizing uptake of bivalent boosters and nirmatrelvir-ritonavir based on age group (65+ years and older) rather than vaccine status would be efficient and have substantial public health impact, but would not address the entire burden of severe COVID-19.

**Disclosures:**

**All Authors**: No reported disclosures

